# Unilateral Resection of the Anterior Medial Temporal Lobe Impairs Odor Identification and Valence Perception

**DOI:** 10.3389/fpsyg.2015.02015

**Published:** 2016-01-08

**Authors:** Stephanie A. Juran, Johan N. Lundström, Michael Geigant, Eva Kumlien, Mats Fredrikson, Fredrik Åhs, Mats J. Olsson

**Affiliations:** ^1^Division of Psychology, Department of Clinical Neuroscience, Karolinska InstitutetStockholm, Sweden; ^2^Unit of Work Environment Toxicology, Institute of Environmental Medicine, Karolinska InstitutetStockholm, Sweden; ^3^Monell Chemical Senses CenterPhiladelphia, PA, USA; ^4^Department of Psychology, University of PennsylvaniaPhiladelphia, PA, USA; ^5^Mental Health Care, Stockholm County CouncilStockholm, Sweden; ^6^Neurology, Department of Neuroscience, Uppsala UniversityUppsala, Sweden; ^7^Department of Psychology, Uppsala UniversityUppsala, Sweden

**Keywords:** anterior medial temporal lobe, amygdalo-hippocampectomy, olfactory perception, odor valence, brain lateralization, temporal lobe epilepsy (TLE)

## Abstract

The anterior medial temporal lobe (TL), including the amygdala, has been implicated in olfactory processing, e.g., coding for intensity and valence, and seems also involved in memory. With this background, the present study evaluated whether anterior medial TL-resections in TL epilepsy affected intensity and valence ratings, as well as free and cued identification of odors. These aspects of odor perception were assessed in 31 patients with unilateral anterior medial TL-resections (17 left, 14 right) and 16 healthy controls. Results suggest that the anterior medial TL is in particular necessary for free, but also cued, odor identification. TL resection was also found to impair odor valence, but not intensity ratings. Left resected patients rated nominally pleasant and unpleasant odors as more neutral suggesting a special role for the left anterior TL in coding for emotional saliency in response to odors.

## Introduction

The medial temporal lobe (TL) is the main host for brain areas involved in both memory, emotional, and olfactory processing in the mammalian brain (van Hartevelt and Kringelbach, 2012; Lehn et al., 2013; Hudry et al., 2014).

Several cortical areas located within the medial TL, among others the piriform cortex, the amygdala, the entorhinal cortex as well as the hippocampal formation, have been linked to olfactory processing due to their close anatomical connectivity to the olfactory receptor neurons (Room et al., 1984; Carmichael et al., 1994). Work on a variety of animal models contributed significantly to our functional understanding of the olfactory neural pathway (Wilson, 2001) and the vast development of functional imaging techniques in the last decade finally succeeded in transferring this knowledge also to human olfaction by showing activation of these areas upon a variety of olfactory tasks (cf. Lundström et al., 2011).

Among all brain structures residing in the medial TL, amygdala has proven strong involvement in olfactory processing. It is located within a monosynaptic projection from the olfactory receptor neurons and has been associated with several aspects of odor processing (as reviewed in Seubert et al., 2013), odor recognition (Jung et al., 2006), odor-association learning (Gottfried et al., 2002), odor intensity coding (Anderson et al., 2003) but also with combined coding of both olfactory intensity and valence (Winston et al., 2005). Due to this vast variety of functional involvement, the specific contribution of amygdala in different olfactory tasks is still difficult to define. Thus, human lesion studies may contribute valuable knowledge because they suggest for example that intensity and quality judgments are functionally separated within the medial TL. Lesions to the medial TL, formed by either resection or reoccurring epileptic activity, impair humans’ ability to assess the identity or quality of odors, while leaving the ability to detect odors and perform odor intensity-scaling tasks intact (Eichenbaum et al., 1983; Jones-Gotman and Zatorre, 1988).

Less is known about the functional relevance of the dense bulbar and piriform projections to the entorhinal cortex and hippocampus (Room et al., 1984). Corresponding to their traditional role in memory processing, they are commonly associated with identification and retrieval of odor qualities (Kjelvik et al., 2012; Lehn et al., 2013). However, emerging data promote the notion that the entorhinal cortex and temporal pole act as an amodal hub, subserving modality-selective regions formation and retrieval of semantic knowledge (Patterson et al., 2007; Baxter, 2009; Suzuki and Baxter, 2009). As such, these areas have been proposed to act as key relay for the retrieval of olfactory memories including recognition of complex perceptual configurations rather than odor processing *per se* (Biella et al., 2007; Kerr et al., 2007; Chapuis et al., 2013).

Investigations in patients suffering from neural insult restricted to medial TL have contributed considerably to better understanding of the functional relevance of these brain areas for olfactory processing. Patients undergoing epilepsy surgery with resection of anterior TL have been shown to have impaired performance for odor discrimination (Eskenazi et al., 1983; Zatorre and Jones-Gotman, 1991), identification (Jones-Gotman and Zatorre, 1988, 1993; Jones-Gotman et al., 1997), odor matching and recognition (Abraham and Mathai, 1983; Eskenazi et al., 1986; Jones-Gotman and Zatorre, 1993; Dade et al., 2002; Buchanan et al., 2003).

Lateralization of olfactory functions has been investigated with different methods, e.g., using monorhinic odor presentation in healthy individuals, utilizing the fact that the olfactory pathway is primarily unilateral. Other studies have investigated non-operated and operated patients with unilateral TLE. Right dominance has been reported in odor matching (Abraham and Mathai, 1983), recognition (Jones-Gotman and Zatorre, 1993; Broman et al., 2001; Olsson and Cain, 2003) and discrimination (Zatorre and Jones-Gotman, 1991) and left dominance in odor memory tasks (Lee et al., 2002; Buchanan et al., 2003) and recently also in odor valence processing (Hudry et al., 2014). Interestingly, the study by Hudry et al. (2014) reported altered valence evaluation (in the sense of a shift toward unpleasant) in left TLE patients in comparison to both right TLE patients and healthy controls. This latter finding corresponds with early reports on left hemispheric dominance for odor valence processing in healthy participants (Royet et al., 2000) but leaves open the question as to what degree epilepsy contributes to such specific left hemispheric impairment of odor valence ratings. We thus wanted to investigate odor valence perception in patients with TLE after resection of the anterior medial TL.

The aim of the current study was twofold. First, we investigated whether anterior medial TL-resection in TLE patients, as compared to healthy controls, affected odor identification, intensity and valence ratings. Second, we attempted to replicate recent reports of left hemispheric dominance in processing of odor valence compared to odor intensity. To this end we compared patients with TLE after unilateral resection of the left and right anterior medial TL. An odor rating task requiring intensity and pleasantness judgments for a group of two pleasant and two unpleasant odors each in weak and strong concentrations was implemented, as well as an identification test of everyday odors that was provided with and wihtout verbal cues. Because we focused on the question of hemispheric dominance in these olfactory tasks, we investigated two patient groups with unilateral right and left lesions using consecutive, unilateral odor stimulation of each nostril. To control for general differences in evaluation of sensory stimuli between TLE patients and healthy controls, a visual gray-scale rating task was included to the procedure.

## Materials and Methods

### Subjects

Forty-five individuals participated in the study, 14 healthy controls (6 male) and 31 patients (13 male) with TLE. All patients had undergone surgery with unilateral anterior medial temporal resection (ATR) including amygdala and hippocampus at Uppsala University hospital (Spencer et al., 1984). In four patients, post-operative MRI examination showed incomplete resection of the amygdala. Time for surgery was on average 6.9 years (*SD* = 4.6) before study participation. Neuropsychological functions were tested and have been reported elsewhere (Åhs et al., 2010, 2013). In the patient group, 28 were right and 5 left-handed, controls were all right-handed. The patient group was subdivided based on side of resection and left- and right ATR subgroups were matched for age, seizure duration, clinical outcome, and neuropsychological performance (Åhs et al., 2010).

All 45 participants (31 patients) completed the first two parts of the study including an odor identification task (cued and free odor identification) and a visual gray-scale rating task. A subgroup of 25 of 31 patients also performed a third task, being an odor intensity and pleasantness rating task including four odors (two pleasant, two unpleasant) in two concentrations each (weak, strong). Demographic data of patient and control group are given in **Table 1**. An independent samples *t*-test was used to compare age between the groups, showing that patient groups did not differ from each other, neither in the whole sample with *N* = 31 [*t*_(29)_ = -0.6, *p* > 0.5] nor in the subsample with *N* = 25 that performed the odor intensity and pleasantness tasks [*t*_(23)_ = -0.8, *p* > 0.3]. However, the group of 14 healthy controls was younger than the patients [*N* = 31; *t*_(43)_ = -3.6, *p* < 0.01].

**Table 1:** Mean age of participant groups in the odor identification and odor rating task.

	Odor identification		Odor rating	
Group	*N*	Mean (*SD*)	*N*	Mean (*SD*)
Left ATR	19	44.6 (10.7)	14	43.2 (7.4)
Right ATR	12	47.2 (11.1)	11	46.5 (10.8)
Both ATR	31	45.5 (10.5)	25	44.6 (9.3)
Control	14	33.6 (9.17)	14	33.6 (9.17)

*ATR, anterior medial temporal lobe resection; N, number of participants; SD, Standard deviation*.

### Odor Identification Tests

Testing usually lasted about 50 min and took place in a well-ventilated room at Uppsala University. Odor stimuli for the identification task were taken from the “*Sniffin’ Sticks: Identification – Extended Test*” (Hummel et al., 2007). Sixteen available odors, all commonly rated as very familiar, provided in pen-like devices, were grouped into two sets A (orange, leather, cinnamon, peppermint, banana, lemon, licorice, turpentine) and B (garlic, coffee, apple, clove, pineapple, rose, aniseed, fish). Both odor sets were presented monorhinally to all participants with order of odor set and nostril being balanced over participants and over patient groups (resection side) and all odor testing was performed with participants’ eyes closed.

Each participant was presented with both odor sets (A/B) at one nostril each, whereas the other nostril was held closed by the participant. After presentation of the first odor set to the first nostril, odor set and side of exposure were changed, randomized between individuals. Each odor was presented for about 4 s in 2–3 cm distance from the exposed nostril. “Free identification” of the odor (FID) was assessed first. Following their response (or acknowledgment that they did not know the answer), participants chose from four possible answers (translated to Swedish from the “*Sniffin’ Sticks”* test) that were presented simultaneously during 5 s as a “cued identification” (CID) test. Four of these answer alternatives where replaced with similar ones to prevent priming effects for upcoming odor presentations. Answers from FID and CID were coded with one point in case of correct identification and zero for no or clearly incorrect answer. Half a point, was given for correctly identified category (e.g., “fruit” for orange) in FID test. Sum of correct answers was analyzed using analysis of variance (ANOVA) including the between-group factor *Group* (left resected, right resected, control) and the within-group factor *Presentation side* (left, right nostril). Fisher’s *Least Significant Difference* was used for *post hoc* test.

### Odor Rating Tests

Two pleasant (citral and peach) and unpleasant (valeric acid, butyric acid) odorants were purchased from Sigma–Aldrich and prepared in weak and strong concentrations by blending to 10 ml with mineral oil. Volume concentrations, calculated as odorant volume divided by target volume 10 ml, are given in brackets for weak and strong blends, respectively: citral (0.01, 1), peach (0.01, 0.5), valeric acid (0.02, 0.1), and butyric acid (0.0067, 0.1). Stimuli were prepared in 160 ml wide mouth (62 mm) opaque glass jars with screw cap and presented by experimenter in 3–4 cm distance from the participant’s nostril for about 4 s. A series of all eight odors was presented monorhinally and each odor was given twice to each nostril of each participant (8 × 2 × 2 stimulations) with 1 min break between concentration series at same nostril and longer breaks between presentations at different nostril. All odor testing was performed with participants’ eyes closed. This exposure procedure was pre-tested in a pilot study in order to warrant iso-intensity of odor blends within the respective groups ‘weak’ and ‘strong’ and to optimize inter-stimulus interval to allow for headspace saturation with the given odorant and glass jar volumes. Odor series was: valeric acid -weak, -strong; peach -weak, -strong; citral -weak, -strong; and butyric acid -weak, -strong with second presentation in reversed order. After each odor presentation, participants used two nine-point scales, one ranging from nothing (0) to maximal (9) to rate odor intensity, and the second ranging from very bad (-4) via neutral (0) to very good (+4), to rate odor pleasantness. Intensity and valence data were investigated separately using mixed model designs. In a first step, we compared odor rating performance in healthy participants and whole group of ATR patients, i.e., regardless of side of resection, thus defining the between-group factor *Group* (control, ATR). In a second step, we addressed the question if left and right ATR have different impact on evaluation of odor intensity and valence by comparing both patient groups with each other, thus using the between-group factor *Patient Group* (left ATR, right ATR). The within-group factor *Stimulation Side* (left, right nostril) was included in all analyses. The within-group factor *Concentration* (weak, strong) was included in analyses of intensity ratings whereas the within-group factor *Valence* (pleasant, unpleasant) was included in analysis of pleasantness ratings in order to confirm that our manipulations of odor intensity and pleasantness were perceived accordingly by all groups.

### Visual Gray Scale

Eight squares ranging in shades of gray between 20 and 90% white content (steps of 10%, created in Microsoft’s Powerpoint) were selected as visual stimuli and their grayness was rated by the participants. The task was selected to be independent of both olfactory as well as semantic processing. This task was presented to the participant on a color calibrated computer screen in a self-paced manner. To become acquainted with the stimulus material, participants were shown all eight squares of different grayness simultaneously at beginning of the task, followed by successive presentation of each square for “grayness” rating using a visual nine-point scale ranging from 1 = ‘completely white,’ through 5 = ‘intermediate,’ to 9 = ‘completely black.’ Thus, the participants’ task was to rate square grayness, it was not required to match stimuli with rating scale options. A random series of visual stimuli was presented twice to each participant and mean grayness-ratings given by each participant were analyzed using a mixed model analysis including the between-group factor *Group* (control, left ATR, right ATR).

This study was carried out in accordance with the recommendations of the local ethics committee. All subjects gave written informed consent in accordance with the Declaration of Helsinki.

## Results

### Free Odor Identification and Cued Odor Identification

**FIGURE 1:**
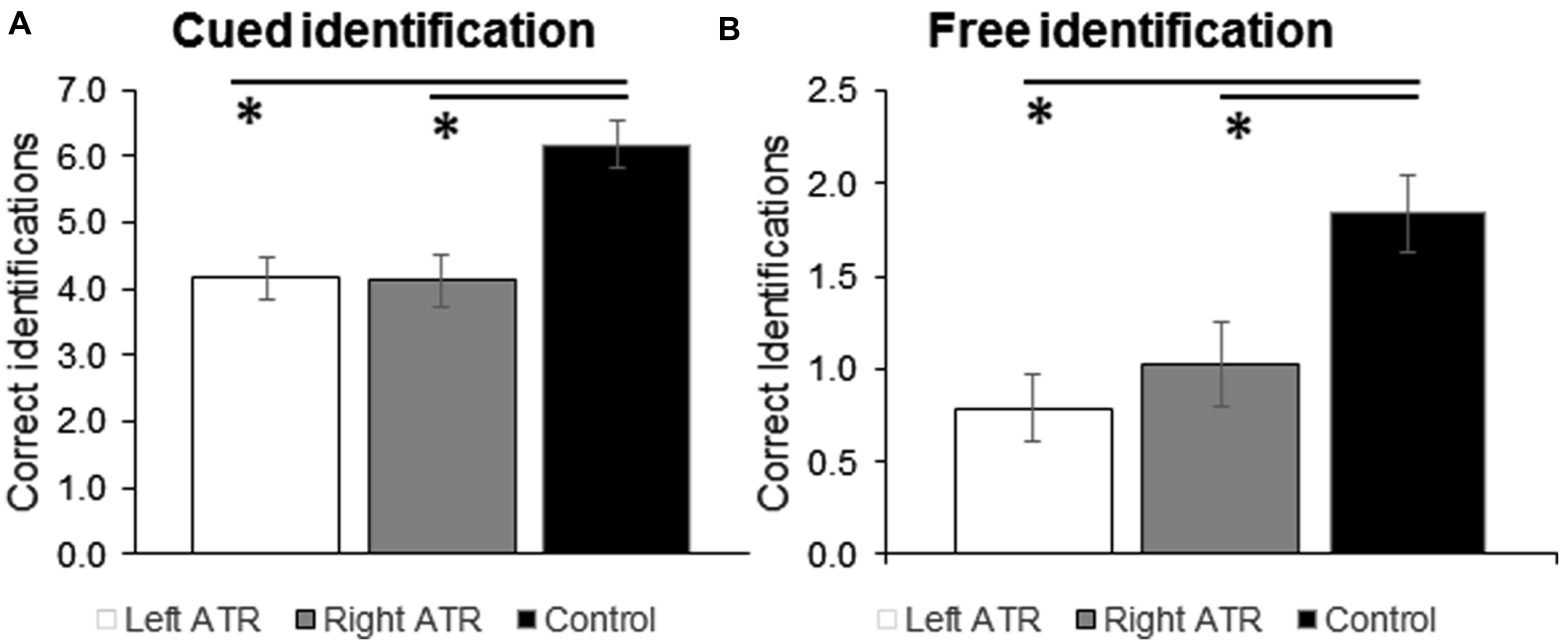
**Mean ratings and standard error of means (SEM) are shown for correct answers given in the cued **(A)** and free odor identification task (B)**. Data are shown separately for the experimental groups left ATR, right ATR, and control. ATR, anterior medial temporal lobe (TL) resection. ^∗^*p* < 0.05 for comparison against control group.

Results from two-way ANOVAs (*Group* × *Stimulation Side*) are given in **Figures 1A,B**. Groups (control, Left or Right ATR) differed in performance in the free [*F*_(2,42)_ = 7.6; *p* = 0.002] and the cued odor identification task [*F*_(2,42)_ = 10.8; *p* < 0.001]. *Post hoc* test revealed that both groups of patients were significantly worse in odor identification than the control group in both FID and CID (all *p* < 0.02). Left and right resected patients, however, did not differ from each other in free (*p* = 0.427) or cued odor identification task (*p* = 0.948). Presentation side did not have a significant effect on performance in the FID or CID task (both *p* > 0.2).

### Odor Intensity

Odor intensity ratings are given in **Table 2**. Mixed Model analysis (*Group* × *Stimulation Side* × *Concentration*) showed a significant main effect for the factor *Concentration* [*F*_(1,1238)_ = 88, *p* < 0.001] indicating that both groups (ATR, controls) correctly differentiated between weak and strong odors by showing significantly lower intensity ratings for the weak as compared to the strong stimuli. No other significant main effects [*Group*: *F*_(1,1238)_ = 1, *p* = 0.3; *Stimulation Side*: *F*_(1,1238)_ = 0.1, *p* = 0.7] and no significant interactions between the three factors were found (all *F*_(1,1238)_ < 0.9, *p* > 0.7). Thus, we can conclude that the group of ATR patients did not differ from controls in perception of odor intensity.

**Table 2:** Mean values and standard error of means (in brackets) are given for odor intensity and grayness ratings for control group (*N* = 14) and patient group (*N* = 25).

	Intensity ratings		Grayness ratings
Group	Strong	Weak	
Control	5.6 (0.1)	4.4 (0.1)	6 (0.19)
Patient	5.5 (0.1)	4.3 (0.1)	
Left ATR	5.4 (0.2)	4.3 (0.2)	6.1 (0.17)
Right ATR	5.7 (0.2)	4.3 (0.2)	6.1 (0.17)

*ATR, anterior medial temporal lobe resection*.

In a second mixed model analysis of odor intensity ratings, we wanted to investigate whether left and right ATR have differential effects on odor evaluation. We therefore focused on comparing only the patient groups with each other. Included to the analysis were the between-group factors *Patient Group* (left ATR, right ATR) and *Concentration* (weak, strong) as well as the within-group factor *Stimulation Side* (left, right nostril), results are given in **Table 2**. As in the first analysis of patient vs. control groups, a significant main effect of *Concentration* [*F*_(1,790)_ = 53.6, *p* < 0.001] was found indicating perceptual discrimination between weak and strong odors. No further significant main effects [*Group*: *F*_(1,790)_ = 0.7, *p* = 0.39; Stimulation Side: *F*_(1,790)_ = 1.9, *p* = 0.16] and no significant interactions between the three factors were found [all *F*_(1,790)_ < 0.8, *p* > 0.3]. These results indicate that resection side did not modulate the perception of odor intensity in left and right ATR patients differently.

### Odor Valence

Mixed model analysis (*Group* × *Valence* × *Stimulation Side*) comparing control group with ATR patients irrespective of resection side showed a significant main effect for the factor *Valence* [*F*_(1,1240)_ = 828,4, *p* < 0.001], thus confirming successful manipulation of odor valence, which was indicated by positive (*M* = 1.3, *SEM* = 0.6) and negative (*M* = -1.3, *SEM* = 0.6) pleasantness ratings for nominally pleasant and unpleasant odors, respectively. Furthermore, a significant main effect of the factor *Group* was found [*F*_(2,1240)_ = 7.8, *p* = 0.005], showing that control group rated odorants as more pleasant than patient group (*M* = 0.13, *SEM* = 0.07 and *M* = -0.12, *SEM* = 0.06, respectively). A significant *Group* × *Valence* interaction [*F*_(1,1240)_ = 24.5, *p* < 0.001] was explained by more extreme un/pleasantness ratings in control subjects that differed significantly from patient ratings for pleasant (*p* < 0.001) but not for unpleasant odors (*p* = 0.13) as tested with pairwise comparisons. No other significant main effect [*Stimulation Side: F*_(1,1240)_ = 0.4, *p* = 0.5] or other significant interaction between the three factors were found [both *F*_(1,1240)_ ≤ 0.15, *p* ≥ 0.7].

**FIGURE 2:**
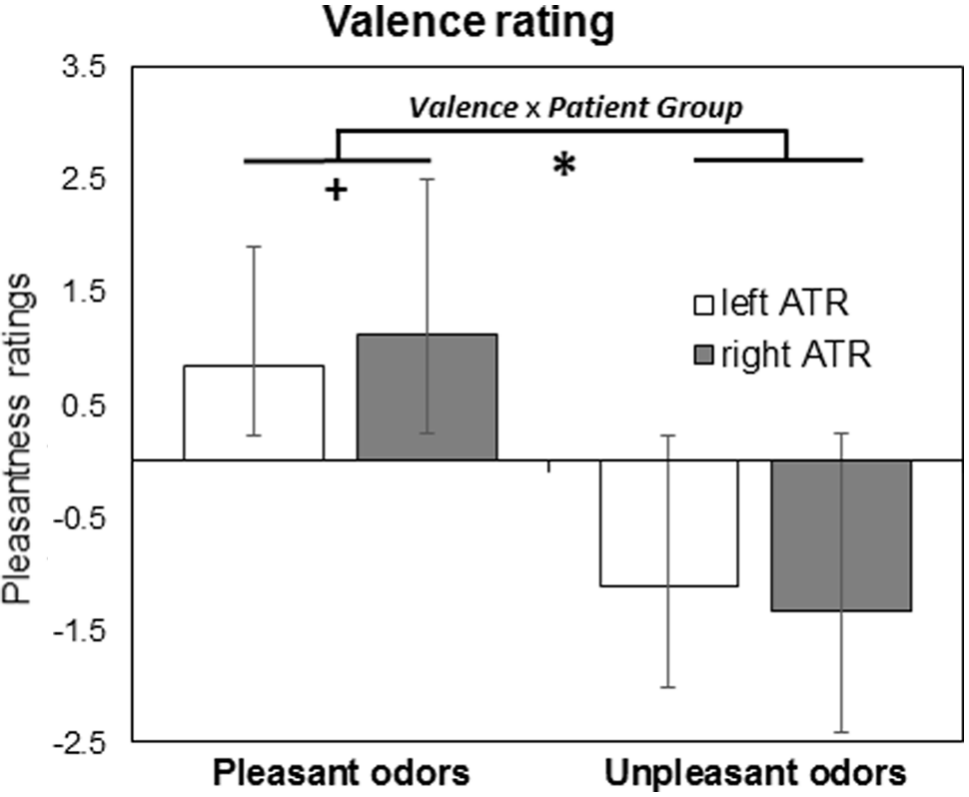
**Results from valence rating task are given as mean and standard error of means for patient groups with left (*N* = 14) and right (*N* = 11) anterior medial temporal lobe resection (ATR)**. ^∗^*p* < 0.05 for Valence × Patient Group interaction, ^+^*p* < 0.1 for pairwise comparisons.

In a second analysis, we investigated whether left and right ATR patient groups differed in odor valence perception. Results of a three-way Mixed Model analysis (*Patient Group* × *Valence* × *Stimulation Side*) are given in **Figure 2**, revealing a significant effect of *Valence* [*F*_(1,792)_ = 332, *p* < 0.001] but not *Patient Group* [*F*_(1,792)_ = 0.1, *p* = 0.8] or *Stimulation Side* [*F*_(1,792)_ = 1.6, *p* = 0.2]. A significant *Patient Group* × *Valence* interaction [*F*_(1,792)_ = 4.3, *p* = 0.038, **Figure 2**] reflected weaker differentiation between positive and negative odors in left ATR group irrespective of stimulation side. Pairwise comparison between left and right ATR groups showed a trend for pleasant odors (*p* = 0.09) but not for unpleasant odors (*p* = 0.21). No other significant interaction was found [*F*_(1,792)_ < 0.4, *p* > 0.5].

### Visual Gray Scale

Data from gray scale test are shown in **Table 2**. Performance showed reasonable test-retest reliability (correlating performance in first and second half of visual gray scale test) ranging between *r* = 0.71 and *r* = 0.99 in patient groups and between *r* = 0.78 and *r* = 0.99 in the control group, thus indicating that patient and healthy controls were as reliable in their use of numbers to rate their perception. Grayness ratings were not significantly modulated by the factor *Group* as shown by the mixed model analysis [*F*_(2,36)_ = 0.03, *p* = 0.97].

## Discussion

Aims of this study were to investigate whether anterior medial TL-resection in TLE patients affected odor processing and to specifically estimate the role of left ATR in odor evaluation. Our results showed impaired odor identification and odor valence ratings, but not odor intensity ratings, in ATR patients as compared to healthy controls and confirmed special deficit in odor valence rating in patients with left ATR.

The study showed that free odor identification was about twice as good, and cued odor identification about 50% better in healthy controls as compared to ATR patients. With reference to normative data on cued odor identification (Hummel et al., 1997), this difference is larger than what would be expected from age difference between patients and controls alone. CID accuracy levels in control group lie at 75% which is below 85% reported by Hummel et al. (1997) but corresponds with accuracy levels reported by Olsson and Cain (2003) who also used monorhinal odor presentation.

Odor intensity ratings did not differ between control and patient groups (**Table 2**), thus confirming isointense perception between groups. *Stimulation Side* did not modulate control group performance in any of the olfactory tasks. Earlier studies have reported inconsistent results using monorhinic odor presentation in healthy volunteers. Broman et al. (2001) found no difference for odor identification whereas Herz et al. (1999) found left side advantage. Herz et al. (1999) also observed higher pleasantness ratings following right side presentation. Such findings from monorhinic odor presentations are often interpreted as related to hemispheric dominance. However, human imaging studies investigating olfactory hemispheric dominance seem to indicate a special role for the left hemisphere and especially left amygdala in valence perception (Zald and Pardo, 1997; Royet and Plailly, 2004). We will further discuss hemispheric lateralization in odor valence perception when discussing findings in our left ATR patient group.

With regard to valence ratings, healthy controls rated odors (across positive and negative odors) as more pleasant than ATR patients. The lower valence ratings, in absence of lower intensity ratings after ATR, may reflect compromised emotional processing as well as faulty semantic processing. Indeed, it has been demonstrated that valence ratings of familiar or namable odors to a great extent are dependent on the name given to the odor object rather than the odor itself (Djordjevic et al., 2008). Thus, odor identification deficits in ATR patients and related shortage of semantic odorant information may have contributed to observed differences in odor valence perception. Another plausible explanation to differences in valence ratings between patients and controls concern rating tendencies. To control for individual differences in rating tendencies, participants rated grayness of visual stimuli. No group differences in consistency or level of ratings could be detected, thereby suggesting that the observed difference in valence ratings reflects a difference also in valence experience rather than just rating behavior.

Comparison between patient groups showed that left ATR group perceived odorants as more neutral than right ATR group. Reports of hemispheric lateralization of odor valence processing are far from consistent but tend to support left dominance (Zald, 2003; Royet and Plailly, 2004). Such left laterality seems especially true for amygdala activation, which correlates with odorant aversiveness (Zald and Pardo, 1997) and thus, our data could be interpreted as reflecting impaired emotional odorant processing. Alternatively, it has been suggested that odor valence ratings are dominated by processing in the left TL by virtue of its dependence on semantic representation of the odor object (Royet et al., 1999, 2001). However, the more neutral valence ratings in left ATR, as compared to right ATR, was not paralleled by worse odor identification, thus indicating that the observed lateralization of valence perception may have been more driven by compromised emotional rather than semantic processing. Altogether these results extend recent findings of an extensive study of TLE patients (Hudry et al., 2014) to patients after ATR. Hudry et al. (2014) report lower (in the direction of less pleasant) valence in left TLE across pleasant and unpleasant odors, which is paralleled by reduced pleasantness ratings following left nostril stimulation when comparing healthy controls with patient groups regardless of TLE lateralization. Interestingly, in the latter comparison Hudry et al. (2014) report the same pattern of more neutral pleasantness ratings for patient group irrespective of TLE lateralization. Thus, both the Hudry et al. (2014) study and our findings suggest left TL involvement in odor valence processing. Possible reasons for differences between these two studies may be related to the fact that patients in our study were assessed post-surgery and also several years after resection. The olfactory system has a large plasticity, from the receptor to the cortical level, thus the mainly perceptual process of odor intensity ratings may have recovered due to a functional reorganization, similarly to what was recently demonstrated in hippocampal processing in patients with TLE (Banks et al., 2012).

## Conclusion

This study shows that epilepsy patients with anterior medial TL resection have compromised olfactory cognition. This is particularly true for free odor ID but also cued odor ID. In addition, an altered valence perception does not seem to depend on a general change in rating behavior or odor intensity perception. The left anterior medial lobe shows a special role for valence perception in line with previous findings. According to our pattern of results, patients with left ATR experienced odors to be less emotionally salient, possibly reflecting an absent or deficient left amygdala.

## Author Contributions

Contribution of authors were as follows: concept and design of work, acquisition, analysis of data: EK, MF, MG, MO. Analysis, interpretation of data, drafting, and revising of the final manuscript: SJ, JL, FÅ, MO.

## Conflict of Interest Statement

The authors declare that the research was conducted in the absence of any commercial or financial relationships that could be construed as a potential conflict of interest. In special this includes that we or our institutions did not receive payment or services from a third party for any aspect of the submitted work at an times.
